# Trends in incidence, prevalence, and survival of primary liver cancer in the United Kingdom (2000–2021)

**DOI:** 10.1093/eurpub/ckaf153

**Published:** 2025-11-10

**Authors:** Berta Cuyàs, Edilmar Alvarado-Tapias, Eng Hooi Tan, Asieh Golozar, Talita Duarte-Salles, Antonella Delmestri, Josepmaria Argemi, Wai Yi Man, Edward Burn, Carlos Guarner-Argente, Daniel Prieto Alhambra, Danielle Newby

**Affiliations:** Department of Gastroenterology, Hospital de la Santa Creu i Sant Pau, Universitat Autònoma de Barcelona, Barcelona, Spain; Medicine Department, Autonomous University of Barcelona (UAB), Barcelona, Spain; Centre for Biomedical Research in Liver and Digestive Diseases Network (CIBERehd), Instituto de Salud Carlos III, Madrid, Spain; Department of Gastroenterology, Hospital de la Santa Creu i Sant Pau, Universitat Autònoma de Barcelona, Barcelona, Spain; Medicine Department, Autonomous University of Barcelona (UAB), Barcelona, Spain; Centre for Biomedical Research in Liver and Digestive Diseases Network (CIBERehd), Instituto de Salud Carlos III, Madrid, Spain; Centre for Statistics in Medicine, Nuffield Department of Orthopaedics, Rheumatology and Musculoskeletal Sciences, University of Oxford, Oxford, United Kingdom; Nemesis Health, New York, NY, United States; OHDSI Center at the Roux Institute, Northeastern University, Boston, MA, United States; Fundació Institut Universitari per a la recerca a l’Atenció Primària de Salut Jordi Gol i Gurina (IDIAPJGol), Barcelona, Spain; Department of Medical Informatics, Erasmus University Medical Centre, Rotterdam, The Netherlands; Centre for Statistics in Medicine, Nuffield Department of Orthopaedics, Rheumatology and Musculoskeletal Sciences, University of Oxford, Oxford, United Kingdom; Centre for Biomedical Research in Liver and Digestive Diseases Network (CIBERehd), Instituto de Salud Carlos III, Madrid, Spain; Liver Unit, Clinica Universidad de Navarra, DNA & RNA Medicine Program, CIMA University of Navarra, Pamplona, Spain; Division of Gastroenterology Hepatology and Nutrition, University of Pittsburgh, Pittsburgh, PA, United States; Centre for Statistics in Medicine, Nuffield Department of Orthopaedics, Rheumatology and Musculoskeletal Sciences, University of Oxford, Oxford, United Kingdom; Centre for Statistics in Medicine, Nuffield Department of Orthopaedics, Rheumatology and Musculoskeletal Sciences, University of Oxford, Oxford, United Kingdom; Department of Gastroenterology, Hospital de la Santa Creu i Sant Pau, Universitat Autònoma de Barcelona, Barcelona, Spain; Centre for Statistics in Medicine, Nuffield Department of Orthopaedics, Rheumatology and Musculoskeletal Sciences, University of Oxford, Oxford, United Kingdom; Department of Medical Informatics, Erasmus University Medical Centre, Rotterdam, The Netherlands; Centre for Statistics in Medicine, Nuffield Department of Orthopaedics, Rheumatology and Musculoskeletal Sciences, University of Oxford, Oxford, United Kingdom

## Abstract

Primary liver cancer (PLC) remains a global health challenge. Understanding trends in the disease burden and survival is crucial to inform decisions regarding screening, prevention, and treatment. Population-based cohort study using UK primary care data from the Clinical Practice Research Datalink (CPRD) GOLD (2000–2021), replicated in CPRD Aurum. Crude and age-standardized incidence rates (IRs), crude period prevalence (PP), and survival at 1, 5, and 10 years were calculated, and stratified by age, sex, and diagnosis year. The crude IR of PLC was 4.56 (95% CI 4.42–4.70) per 100 000 person-years between 2000 and 2021, with an increase over time across age and sex strata. Sex-specific IR for males was higher than females, 6.60 (95% CI 6.36–6.85) vs. 2.58 (95% CI 2.44–2.74) per 100 000 person-years. Age-standardized IR showed identical trends. Crude PP showed a seven-fold increase over the study period, with PP 0.02% (95% CI 0.019%–0.022%) in 2021, and a 2.8-fold higher PP in males. Survival at 1, 5, and 10 years after diagnosis was 41.7%, 13.2%, and 7.1%, respectively, for both sexes. One-year survival increased only in men, from 33.2% in 2005–2009 to 49.3% in 2015–2019. Over the past two decades, there has been a substantial increase in the number of patients diagnosed with PLC. Despite a slight improvement in median and one-year survival in men, prognosis remains poor. To improve the survival of PLC patients, it is necessary to understand the epidemiological changes and address preventable risk factors associated with liver disease and promote early detection and access to care.

## Introduction

Primary liver cancer (PLC) is the sixth most common cancer and the third leading cause of cancer death worldwide [1], with hepatocellular carcinoma (HCC) accounting for approximately 90% of PLC cases [2].

The incidence and mortality of PLC are growing worldwide, with approximately 906 000 new cases and 830 000 deaths in 2020. Incidence remains highest in Eastern Asia and Northern Africa, but it is increasing in different parts of Europe, Americas, and Oceania [3]. PLC is also among the top five causes of cancer mortality in several European and Western Asian countries [4], with predicted cases and mortality rising by over 50% in the next 20 years [1]. The UK is expected to see one of the highest increases in incidence over the next decade [5]. Despite survival improvements over previous decades due to advancements in systemic treatment, surgical techniques, and the shift towards multidisciplinary teams, survival remains poor even in high-income countries, with one- and five-year relative survival estimates around 40% and 10%, respectively [4, 6].

The main risk factors for PLC are gradually changing over time and between populations. While direct-acting antiviral therapy has substantially reduced the global risk from Hepatitis C virus (HCV) since 2017, there is an increasing rate of alcohol use, obesity, diabetes, and metabolic syndrome. As a result, alcohol-associated liver disease (ALD) and metabolic dysfunction-associated steatotic liver disease (MASLD) now represent the main risk factors, with MASLD present in up to 20% of the population, with MASLD-related HCC rising sharply with over 60% of HCC cases showing features of metabolic syndrome [7].

With rising risk factors such as obesity, diabetes, and alcohol consumption, comprehensive assessments of PLC trends in the UK are increasingly important. Understanding trends in incidence, prevalence, and survival is crucial for guiding decisions on screening, prevention, and treatment. This study is the first to use primary care data, offering new insights into the evolving landscape of PLC in the UK.

## Methods

### Data sources and study design

We conducted a population cohort study using primary care data from the UK. People with a PLC diagnosis and a background cohort (denominator population) were identified from Clinical Practice Research Datalink (CPRD) GOLD (July 2022). We additionally used CPRD Aurum for comparison. These databases contain pseudo-anonymized patient-level information on demographics, lifestyle data, clinical diagnoses, prescriptions, and preventive care contributed by UK general practitioners. CPRD GOLD contains data from across the UK whereas Aurum includes only England. The use of CPRD data was approved by CPRD’s Research Data Governance process (22_001843). Both primary care databases are broadly representative of the UK population [8] and were mapped to the Observational Medical Outcomes Partnership (OMOP) Common Data Model [9].

### Study participants and time at risk

Participants were aged 18+ with at least 1 year of prior history. For incidence and prevalence analysis, the study cohort included individuals present in the databases from 1 January 2000. Follow-up continued until the practice stopped contributing to the database, the patient left the practice, death, or end of the study (31 December 2021 for GOLD, 31 December 2019 for Aurum). For survival analysis, individuals with a newly diagnosed cancer were included and followed from diagnosis to either death, practice stopped contributing to the database, patient left the practice, or end of the study period. Patients whose death and cancer diagnosis occurred on the same date were excluded.

### PLC and mortality definitions

We used Systematized Nomenclature of Medicine—Clinical Terms (SNOMED CT) diagnostic codes to identify PLC events. Diagnostic codes related to intrahepatic cholangiocarcinoma, non-malignant cancer or metastasis (apart from prevalence analysis) or melanoma, and lymphoma in the liver were excluded. Clinicians with expertise in oncology, primary care, and real-world data reviewed the PLC codelist. Supplementary Information S1 contains the clinical codelistis, with HCC being the most frequent PLC type. For sensitivity analysis, an additional PLC definition including intrahepatic cholangiocarcinoma clinical codes was created. OMOP-based computable phenotypes and analytical code are available on Github to enable reproducibility (https://github.com/oxford-pharmacoepi/EHDENCancerIncidencePrevalence). For overall and annual crude incidence rates (IRs) and annual prevalence, all PLC events in the study period were included. For survival analyses, mortality was defined as all-cause mortality.

### Statistical methods

The population characteristics were summarized using median and interquartile range (IQR) for continuous variables and counts and percentages for categorical variables.

For crude incidence, the number of events, the observed time at risk, and the IR per 100 000 person-years were summarized along with 95% confidence intervals (95% CIs). Annual IRs were calculated as the number of incident PLC cases as the numerator and the recorded number of person-years in the general population within that year as the denominator whereas overall incidence was calculated from 2000 to 2021.

Age-standardized IRs were calculated using the 2013 European Standard Population (ESP2013) to account for potential changes in population age structure over time. The ESP2013 provides predefined age distribution in five-year age bands; therefore, we collapsed these to obtain distributions for 10-year age bands used in this study. We used the age distribution of 20–29 years from ESP2013 for age-standardization as age distributions were not available for 18–29 years age band used in this study. IRs were also calculated for PLC including intrahepatic cholangiocarcinoma cases as sensitivity analysis for CPRD GOLD.

Period prevalence (PP) was calculated on 1 January for the years 2000–2021, with the number of patients fulfilling the case definition for PLC as the numerator and eligible participants in the respective years as the denominator. The number of events and prevalence (%) were summarized along with 95% CI.

For survival analyses, we used the Kaplan–Meier method to estimate overall survival for PLC from observed survival times with 95% CI. We estimated the median survival and survival at 1, 5, and 10 years after diagnosis. Survival was also calculated for PLC including intrahepatic cholangiocarcinoma cases as sensitivity analysis for CPRD GOLD.

All results were stratified by database, age (10-year age bands apart from the first and last age bands which were 18–29 years and 90+ years, respectively) and sex. For survival analysis, we additionally stratified by calendar time of cancer diagnosis (2000–2004, 2005–2009, 2010–2014, 2015–2019, and 2020–2021) allowing a maximum of 5 years follow-up from cancer diagnosis. To avoid re-identification, we do not report results with fewer than five cases.

For Aurum, the same statistical analyses were performed using data from 1 January 2000 to 31 December 2019 to compare with GOLD, except the calendar time stratification which was only performed in GOLD.

The statistical software R version 4.2.3 was used for analyses. For calculating incidence and prevalence, we used the Incidence Prevalence R package [10].

## Results

### Patient populations and characteristics

Overall, there were 11 388 117 eligible patients with at least 1 year of prior history identified from January 2000 to December 2021 in CPRD GOLD. Attrition tables can be found in Supplement S2.

We identified 3999 patients with PLC in CPRD GOLD. Patient characteristics are shown in Table 1. Patients were mostly male (71%), with a median age of 71 (IQR 62–77) years. The largest age group was 70–79 years (33.4%), for both males and females, similar to Aurum (Supplement S3). Males had higher prevalence of chronic liver disease, ALD, hemochromatosis, diabetes, and smoking, while females had more autoimmune hepatitis. HCV and NAFLD prevalence were similar across sexes.

**Table 1.** Baseline characteristics of primary liver cancer patients at diagnosis stratified by sex from CPRD GOLD

Sex	Male	Female
Number of patients	2848	1151
Age in years, median (IQR)	70 (62–77)	73 (64–80)
Age groups in years, *N* (%)		
18–29	11 (0.4%)	<5
30–39	18 (0.6%)	9 (0.8%)
40–49	97 (3.4%)	41 (3.6%)
50–59	411 (14.4%)	149 (12.9%)
60–69	811 (28.5%)	260 (22.6%)
70–79	952 (33.4%)	385 (33.4%)
80–89	512 (18.0%)	271 (23.5%)
90+	36 (1.3%)	33 (2.9%)
Prior history in days, median (IQR)	3977 (2189–5642)	3943 (2110–5642)
Comorbid conditions (any time prior)		
Chronic liver disease, *N* (%)	663 (23.3%)	176 (15.3%)
Recorded risk factors for PLC, *N* (%)		
Alcoholic liver disease (ALD)	385 (13.5%)	52 (4.5%)
Hepatitis C	91 (3.2%)	25 (2.2%)
Hepatitis B	20 (0.7%)	<5
Non-alcoholic fatty liver disease (NAFLD)	80 (2.8%)	26 (2.3%)
Hemochromatosis	90 (3.2%)	9 (0.8%)
Autoimmune hepatitis	14 (0.5%)	37 (3.2%)
Other recorded risk factors, *N* (%)		
Hypertensive disorder	850 (29.8%)	349 (30.3%)
Diabetes	851 (29.9%)	215 (18.7%)
Hyperlipidaemia	209 (7.3%)	99 (8.6%)
Obesity	313 (11.0%)	102 (8.9%)
Smoking status (any time 5 years prior), *N* (%)		
Current/former smoker	806 (28.3%)	270 (23.0%)
Nonsmoker	954 (33.5%)	559 (48.6%)
Missing/no records	1088 (38.2%)	322 (28.0%)

### IRs stratified by calendar year, age, and sex

**Figure 1.**
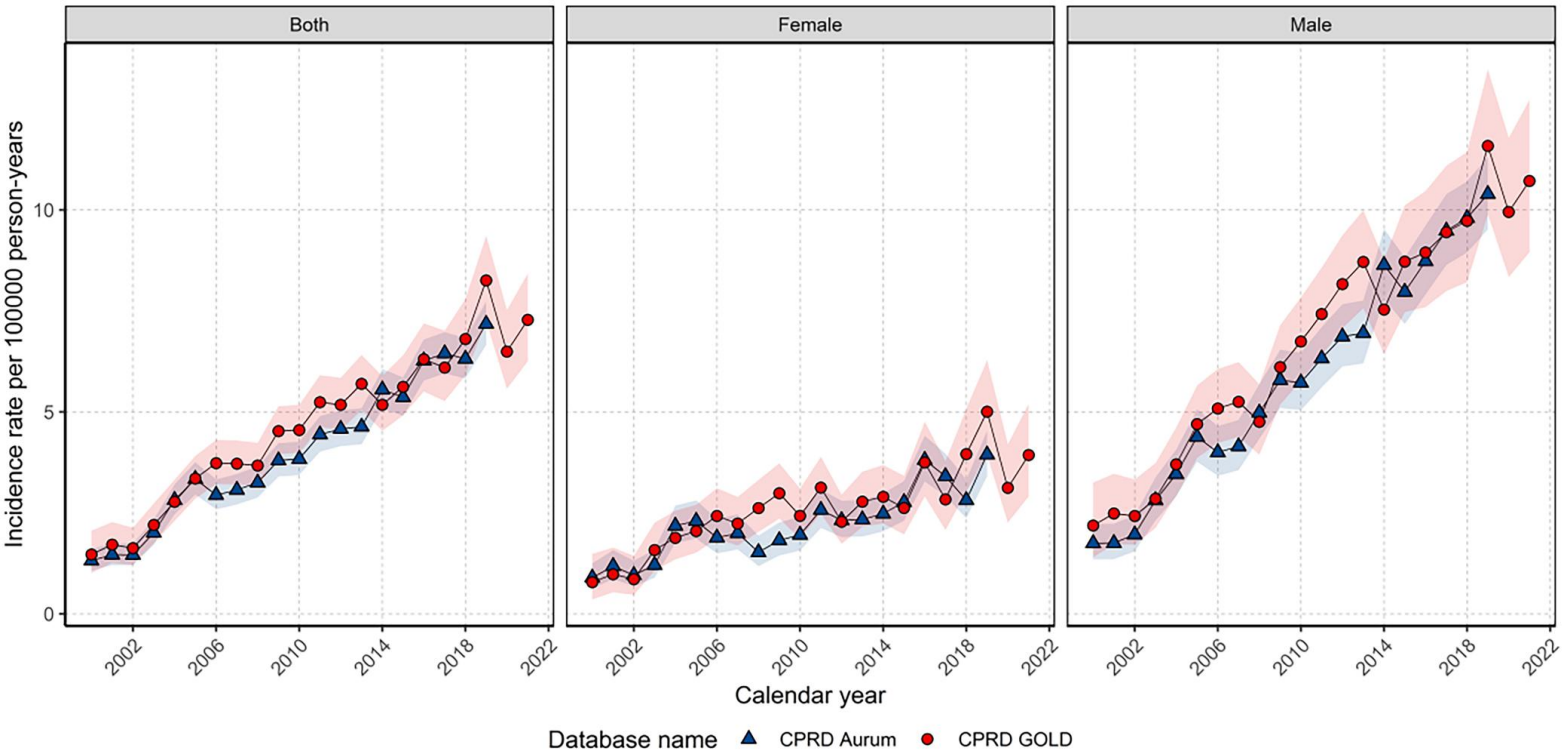
Crude annual incidence rates for PLC from 2000 to 2021 stratified by database and sex.

Unless otherwise stated, IRs are presented as crude rates to reflect the actual burden of disease. The overall crude IR of liver cancer in 2000–2021 was 4.56 (95% CI 4.42–4.70) per 100 000 person-years. Sex-specific IRs were 2.58 (95% CI 2.44–2.74) for females and 6.60 (95% CI 6.36–6.85) for males per 100 000 person-years, with similar results in Aurum. Annualized IRs increased from 2000 to 2019 for the whole population and both sexes, with males having higher rates (Fig. 1). For GOLD, IR dropped in 2020 before recovering in 2021. Age-standardized IRs for GOLD for both sexes showed similar trends (Supplement S4) to crude rates. Sensitivity analysis showed similar age-standardized rates and trends between PLC excluding or including intrahepatic cholangiocarcinoma cases (Supplement S5). Comparison with national cancer registry age-standardized IRs from England showed similar trends but lower age-standardized rates in this work (Supplement S6). Full results are available in a user-friendly interactive web application: https://dpa-pde-oxford.shinyapps.io/LiverCancerIncPrevSurvShiny/. Overall crude IRs were higher with increasing age up to 80–89 years. Those aged 18–29 had the lowest overall IRs (0.09, 95% CI 0.05–0.15) per 100 000 person-years, whereas those aged 80–89 had the highest (17.7, 95% CI 16.4–18.9) with similar or slightly lower IRs in Aurum (Supplement S7).

**Figure 2.**
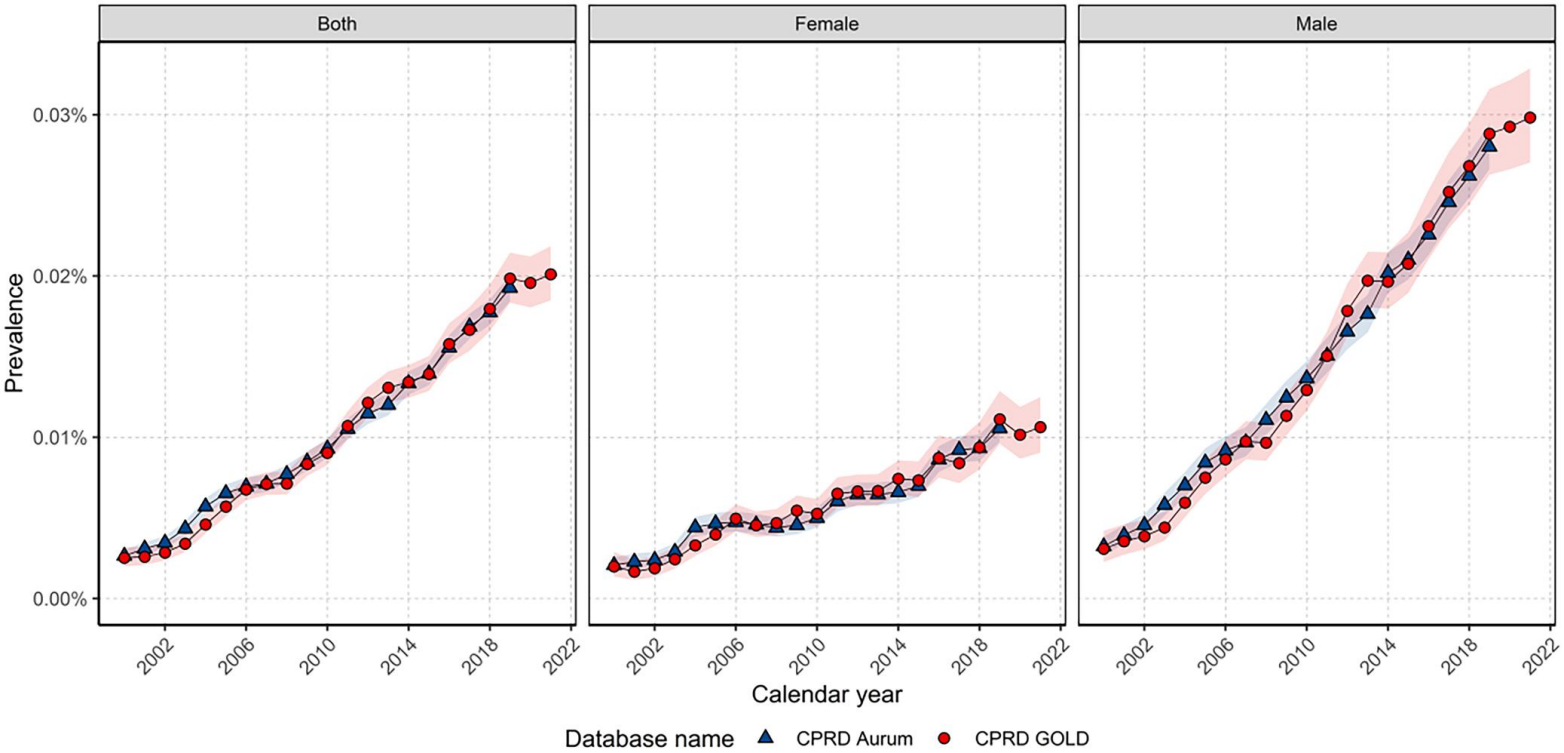
Crude annual prevalence from 2000 to 2021 for whole population and stratified by sex.

Annualized IRs for each age group (Supplement S8) show IRs have increased over the study period for ages 40–89 years. For other age groups data were insufficient to assess trends. Stratification on both sex and age showed similar trends to Fig. 2 for both sexes (Supplement S9). Males had higher IRs than females across the study period, with the gap widening over the study period.

### Prevalence for study population with database, age, and sex stratifications

In 2021, crude PP for PLC in GOLD was 0.020% (0.018%–0.022%) and 2.72-fold higher for males than females. Since 2000, PP increased 6.66-fold, with a 10-fold rise in males compared to 5.5-fold in females. Similar PP was observed from 2000 to 2019 across both databases (Fig. 2).

When stratifying by age group, PP in 2021 was highest in those aged 70–89 years (0.07%). PP increased over the study period for all age groups across both databases except for those aged 40–49 in GOLD where little change occurred between 2011 and 2021 (Supplement S10). Stratification on both sex and age group showed similar trends with females driving the increase in PP in those aged 40–49 years in Aurum (Supplement S11).

### Overall survival rates for cancer population with age, sex, and calendar year stratification

There were 3892 patients with 3047 (78.3%) deaths over the study period in GOLD with a median follow-up of 0.58 (IQR 0.17–1.58) years. Median survival was 0.70 (95% CI 0.65–0.74) years in GOLD and slightly higher in Aurum (∼0.80 years, 95% CI 0.75–0.84) (Supplement S12). Survival was identical between different case definitions of PLC with and without cholangiocarcinoma (Supplement S13). One, 5 and 10 years survival rates were 41.7%, 13.2%, and 7.1% for both sexes in GOLD and slightly higher in Aurum (Supplement S14).

Females had slightly lower median survival than males across both databases, in GOLD 0.62 (95% CI 0.56–0.69) years vs. 0.74 years (95% CI 0.68–0.81). Median survival generally declined from age 40 onwards for both databases (Supplement S15). Short-term survival decreased with age from 30 to 39, with the lowest rates in those aged 90 years and older (Table 2). To investigate if survival has changed over time in GOLD, we stratified by calendar time of cancer diagnosis in five-year age windows for males and females (Supplement S16). Overall, median survival increased from 0.45 (95% CI 0.37—0.65) years for those diagnosed in 2000–2004 to 0.91 (95% CI 0.78–1.00) years for those in 2015–2019. However, this increase was only observed in males: 0.43 (95% CI 0.29–0.60)–0.98 (95% CI 0.86–1.10) years. No clear median survival trends emerged by age groups over time, except for those aged 60–69 years, where median survival rose from 0.63 years for those diagnosed 2005–2009 to 1.33 years for those in 2015–2019 (Supplement S17).

**Table 2.** Survival rates of PLC from 2000 to 2019 for CPRD Aurum and 2000–2021 for GOLD stratified by database and age group

Age group	Survival (%)		
	One-year	Five-year	10-year
Aurum			
18–29	62.9 (47.6–83.0)	46 (30.3–69.9)	–
30–39	65.7 (55.7–77.3)	51.9 (41–65.7)	46.7 (34.1-63.9)
40–49	60.3 (54.9–66.3)	35.8 (30–42.7)	25.5 (19.5–33.3)
50–59	53.3 (50.3–56.5)	22.4 (19.6–25.7)	17.1 (14.1–20.7)
60–69	50.7 (48.4–53.1)	19.1 (17.0–21.4)	10.3 (8.3–12.8)
70–79	42.4 (40.3–44.5)	12.4 (10.8–14.2)	5.3 (3.9–7.2)
80–89	33.9 (31.2–36.8)	6.9 (5.21–9.11)	1.9 (0.7–5.2)
90+	21.6 (15.6–30)	1.6 (0.26–10.2)	–
GOLD			
18–29	31.8 (14.2–70.8)	–	–
30–39	62.5 (45.7–85.5)	44.2 (26.7–73.3)	–
40–49	54.9 (47.1–64.1)	29.1 (21.5–39.3)	26.5 (19.1–36.8)
50–59	48.4 (44.3–52.9)	20.3 (16.8–24.7)	12.5 (9.0–17.4)
60–69	47.8 (44.9–51.0)	16.0 (13.6–18.9)	9.2 (6.9–12.4)
70–79	37.7 (35.1–40.5)	10.1 (8.3–12.3)	3.6 (2.3–5.7)
80–89	28.1 (25.0–31.6)	5.0 (3.3–7.6)	0.7 (0.1–4.3)
90+	30.3 (20.6–44.6)	3.1 (0.5–19.6)	–

One-year survival improved from 35.6% (95% CI 30.5–41.5) in 2000–2004 to 46.7% (95% CI 43.8–49.9) in 2015–2019. Stratification by sex showed one-year survival increased in males only, from 33.2% (95% CI 27.2–40.4) to 49.3% (95% CI 45.9–53.1). For five-year survival, there were no overall or sex-specific improvements (Supplement S18). One-year survival improved only in those aged 60–69 years (35.52% [95% CI 26.6–47.5] to 58.2% [95% CI 52.7–64.3]). There were no clear long-term survival trends in other age groups (data not shown).

## Discussion

This study provides a comprehensive descriptive analysis of PLC trends in the UK from 2000 to 2021. Incidence and prevalence increased across sexes and age groups. Males had slightly higher median survival than females, but one-, five-, and 10-year survival rates were similar between sexes. Short-term survival improved only in males, but no increases in long-term survival were observed overall or by sex and age strata.

Crude and age-standardized IRs for PLC reported here are lower but broadly consistent with National Cancer Statistics (NCS) (9.4 per 100 000 person-years, 2016–2018) [6], and other global studies across high socio-demographic index countries [11, 12]. Slight differences may stem from differing study periods (2000–2021 for our data vs. 2016–2018 for NCS), potential incompleteness/misclassification in primary care records and inclusion of PLC subtypes like cholangiocarcinoma in national statistics, which we excluded. However, including intrahepatic cholangiocarcinoma in sensitivity analysis did not alter incidence or survival results. Similarity in temporal trends between crude and age-standardized IRs indicates that changes in age or population structure are unlikely to explain the observed rise in cases.

In terms of secular trends, PLC crude and age-standardized incidence increased for both sexes across the study period, consistent with recent UK studies. Burton *et al.* reported an increase from 4.4 per 100 000 in 1997 to 9.6 in 2017 in the UK, reaching a plateau since 2014 [13], and Liao *et al.* also noted rising age-standardized IRs from 2008 to 2018 [14]. Our study presents updated data, up to 2021, and follows on from several Commissions which highlighted a 400% increase in liver disease mortality since the 1970s, making it the UK’s third leading cause of premature death. Despite their recommendations to reduce liver diseases in the UK [15], the 2021 Lancet Commission stressed the continued rise in liver diseases, particularly from alcohol and obesity, which is concerning as 49% of PLCs are preventable [16]. Possible reasons that explain the increase in PLC incidence in UK could be the following. Firstly, as most PLCs develop in patients with chronic liver disease, changes in the comorbidity profiles over recent decades are likely to play a key role [15, 17]. In the last 10 years, new treatments for HCV have reduced the prevalence of HCV-related HCCs but non-viral risk factors, such as high alcohol consumption and ALD, are rising at an alarming rate [18]. Data from the Health Survey for England revealed that over 60% of UK adults were overweight or obese in 2021, with higher rates in men [19]. The increase in obesity and diabetes is directly related to the rise in MASLD [20], which may contribute to PLC cases. The main risk factors and the underlying liver diseases (ALD, MASLD) related to PLC identified in this study are consistent with the recent data, showing a shift to non-viral risk factors, a trend expected to continue over the next years [2, 13].

Several studies have reported higher IRs in males compared to females in line with our results, in the UK [13, 14, 21] and worldwide [11]. Higher IRs with increasing age are also supported by this work [22]. A decline in IR in 2020 coincided with the COVID-19 pandemic, similar to other studies showing a significant reduction in HCC diagnosis due to disrupted routine healthcare [23]. The pandemic also influenced the access to HCV treatment in the UK, after the increased availability of direct-acting antiviral therapy since 2017, studies reported a decrease of 40.2% between 2019 and 2021 [24], which could reverse some of the progress made in HCV control and influence future PLC rates [11].

One-, five-, and 10-year survival in our study are in line with UK cancer registry and Burton *et al.* [6, 13] as well as other international studies [4]. While other types of cancer have improved survival in high-income countries [1, 25], PLC remains among the lowest in the UK. Cancer survival improves significantly if diagnosed early. However, in the UK adherence to 6-month ultrasound scan surveillance in cirrhotic patients is suboptimal [26], and only three in 10 PLC are detected early [27], which could explain the low survival rates observed. To address this, NHS England has been promoting an early detection liver cancer pilot programme since 2022, to improve outcomes by screening for advanced fibrosis in high-risk communities [28].

Males have a higher PLC burden globally than females [11], likely due to differences in HCC risk factors, screening adherence, and epigenetics and biological factors [11, 29]. Despite known sex differences in PLC incidence, our study showed slightly lower median and one-year survival for females. Cancer Research UK statistics for 2015–2019 align with our results, showing one-year survival of 39.3% for females vs. 49% for males in Scotland, 35.9% vs. 41.8% in England, 32.5% vs. 37.4% in Wales, and 39.8% vs. 41% in Northern Ireland, respectively [30]. However, other studies report better HCC-overall survival in women, possibly due to better surveillance adherence [29]. Furthermore, a US study found younger females had better survival than younger males, a difference not observed in older adults, hypothesizing a role for sex hormones [31]. Additionally, alcohol consumption in females may be underreported and has increased in recent years [32]. Females are more susceptible to alcohol-induced liver injury leading to cirrhosis and HCC risk, though the mechanisms are unclear, and are less likely to use prevention services due to perceived stigma, conflicting personal/family needs, or financial barriers, resulting in delayed diagnosis [33].

Increasing age causes physiological changes to adapt to organ deterioration, which can be key to liver carcinogenesis. A UK study showed that patients with MASLD-associated HCC were older than those with other aetiologies (71.3 years vs. 67.1 years) and their cancers less often detected by surveillance [34]. However, elderly patients have similar treatment success rates to younger patients [35].

Although overall survival has not improved significantly over time, it is noteworthy that the median survival in our study has doubled from 2000–2004 to 2015–2019, reaching nearly eleven months in males. This increase could be linked to the rising disease burden and unchanged mortality rates. However, Ding *et al.* reported significant survival improvements for HCC patients in the US over the past three decades, attributed to advances in early diagnosis and treatments, such as effective systemic therapy [36]. Divergent trends in HCC survival between the UK and the US are likely due to differences in healthcare infrastructure, slower implementation of screening, and treatment strategies.

The lack of substantial survival improvements in our study period may be influenced by several factors. Firstly, despite declining HCV infections rates, the UKHSA 2023 report suggests that nearly three-quarters of chronic HCV cases remain undiagnosed [24], delaying PLC diagnosis. The slight survival improvements in males may result from HCC screening programs mainly targeting chronic liver disease, more frequent in males, leading to earlier diagnosis with better treatment options [4]. PLC diagnosing delays, especially in women, may be due to socio-demographic barriers such as scarce primary care consultation due to family/social limitations. Finally, better long-term survival in the next decade may come from primary prevention measures like HBV vaccination, reducing alcohol and tobacco use, managing obesity and cardio-metabolic factors, and advances in radiological techniques.

The main strength of this study is the use of two large representative data sources covering the UK. CPRD GOLD covers primary care practices from England, Wales, Scotland, and Northern Ireland whereas CPRD Aurum covers England. The consistency of results across databases provides increased generalizability and robustness of our findings. Additionally, the use of a complete study population database for assessing incidence and prevalence avoids the inaccuracies that can arise in cancer registry studies that extrapolate the registry data using national population statistics [37]. The high validity and completeness of mortality data with over 98% accuracy compared to national mortality records [38] enable a thorough examination of the impact of calendar time on overall survival.

Our study has some limitations. Firstly, using primary care data without linkage to a cancer registry could lead to misclassification and delayed recording of cancer diagnoses. However, previous validation studies have shown high accuracy and completeness of cancer diagnoses in primary care records [39]. Secondly, our use of primary care records also precluded us from studying tumour histology, genetic mutations, tumour stage, or cancer therapies, which impact PLC survival. Finally, the main risk factors for PLC, such as chronic liver disease, may be underrepresented in this study, because they might be infrequently reported in large primary care databases or diagnosed concurrently with PLC, thus not being included. The patterns of alcohol consumption can be also underreported, particularly among women [32], as well as other sociocultural factors.

## Conclusions

In summary, the present study shows that the number of people diagnosed with PLC in the UK has substantially increased over the past 20 years, while overall survival remains low. The shift from virus-related to non-viral liver disease highlights the need for more research and resources for conditions like ALD and MASLD among others. Despite therapeutic advances and slight survival improvements, over half of PLC patients are not alive after 1 year. Therefore, public health interventions such as establishing early diagnostic strategies for potential risk factors and improving screening programmes in cirrhotic patients, as well as education campaigns, could be valuable as future strategies in primary care.

## Supplementary Material

ckaf153_Supplementary_Data

## Data Availability

This study is based in part on data from the CPRD obtained under the University of Oxford multi-study licence from the UK Medicines and Healthcare products Regulatory Agency. The data are provided by patients and collected by the NHS as part of their care and support. The interpretation and conclusions contained in this study are those of the author/s alone. Patient-level data used in this study was obtained through an approved application to the CPRD (application number 22_001843) and is only available following an approval process to safeguard the confidentiality of patient data. Details on how to apply for data access can be found at https://cprd.com/data-access. Key points Primary liver cancer (PLC) is a major public health concern, ranking as the third leading cause of cancer-related deaths worldwide. The primary risk factors for PLC are shifting from viral liver diseases to an increasing prevalence of alcohol-associated liver disease (ALD) and metabolic dysfunction-associated steatotic liver disease (MASLD). In the past two decades, PLC incidence and prevalence have risen in the United Kingdom, with slight improvements in median and one-year survival rates among men, though overall prognosis remains poor. This study provides essential insights into the PLC burden, supporting informed decisions on screening, prevention, and treatment strategies. Addressing preventable risk factors, enhancing early detection, and improving access to care are crucial steps towards reducing PLC-related mortality.
